# Exploration of the Hypothalamic-Pituitary-Adrenal Axis to Improve Animal Welfare by Means of Genetic Selection: Lessons from the South African Merino

**DOI:** 10.3390/ani3020442

**Published:** 2013-05-17

**Authors:** Denise Hough, Pieter Swart, Schalk Cloete

**Affiliations:** 1 Department of Biochemistry, Stellenbosch University, Stellenbosch 7602, South Africa; E-Mails: houghdenise@gmail.com (D.H.); pswart@sun.ac.za (P.S.); 2 Department of Animal Sciences, Stellenbosch University, Stellenbosch 7602, South Africa; 3 Institute for Animal Production, Elsenburg, Private Bag X1, Elsenburg 7607, South Africa

**Keywords:** robustness, stress, cortisol, hypothalamic-pituitary-adrenal axis, marker-assisted selection, SNP, sheep, animal welfare, behaviour

## Abstract

**Simple Summary:**

Breeding sheep that are robust and easily managed may be beneficial for both animal welfare and production. Sheep that are more readily able to adapt to stressful situations and a wide variety of environmental conditions are likely to have more resources available for a higher expression of their production potential. This review explores the utilization of one of the stress response pathways, namely the hypothalamic-pituitary-adrenal axis, to locate potential sites where genetic markers might be identified that contribute to sheep robustness. A South African Merino breeding programme is used to demonstrate the potential benefits of this approach.

**Abstract:**

It is a difficult task to improve animal production by means of genetic selection, if the environment does not allow full expression of the animal’s genetic potential. This concept may well be the future for animal welfare, because it highlights the need to incorporate traits related to production and robustness, simultaneously, to reach sustainable breeding goals. This review explores the identification of potential genetic markers for robustness within the hypothalamic-pituitary-adrenal axis (HPAA), since this axis plays a vital role in the stress response. If genetic selection for superior HPAA responses to stress is possible, then it ought to be possible to breed robust and easily managed genotypes that might be able to adapt to a wide range of environmental conditions whilst expressing a high production potential. This approach is explored in this review by means of lessons learnt from research on Merino sheep, which were divergently selected for their multiple rearing ability. These two selection lines have shown marked differences in reproduction, production and welfare, which makes this breeding programme ideal to investigate potential genetic markers of robustness. The HPAA function is explored in detail to elucidate where such genetic markers are likely to be found.

## 1. Introduction

The ever-increasing global population continues to place pressure on improving the “efficiency” of animal production to meet local and global demands. It is, however, a difficult task to improve animal production in commercial practices by means of genetic progress if the environment in which the animals are raised does not support the full expression of their genetic potential [1]. It is therefore important to include robustness-related traits in breeding objectives to such an extent that selection balances genetic change in production potential with the genetic change in environmental sensitivity [2]. The importance of robustness-related traits is perhaps better understood in the description of “robustness” as the ability to combine a high production potential with resilience to stressors [3], which allows for the unproblematic expression of a high production potential in a wide variety of environments [4].

The inclusion of such objectives is particularly necessary in South Africa, where animals are often raised in adverse production environments [5]. These extreme environments, along with climate change and increasing economic pressure, emphasize the importance of considering robustness-related traits in the development of sustainable breeding goals. The inclusion of robustness-related traits in selection criteria is often neglected at the cost of improving animal production only [2,6,7,8,9].

One example where genetic selection based on production traits alone resulted in a reduction in robustness, is the case of the South African Angora goat. Selection for higher fibre production linked to a lower fibre diameter resulted in hypocortisolism and a susceptibility to cold stress, which was ascribed to the inability to increase blood-glucose levels (generate metabolic heat) via the action of cortisol [10,11]. The cause for hypoadrenocortisolism was found to be a genetic mutation for goat cytochrome P450 17α-hydroxylase/17,20-lyase (CYP17) that resulted in three unique CYP17 genotypes [12]. CYP17 is a steroidogenic enzyme that mediates the synthesis of cortisol in the adrenal gland upon stimulation of the hypothalamic-pituitary-adrenal axis (HPAA). Differences in the activity of the CYP17 isoforms resulted in a reduced ability of Angora goats to produce cortisol in response to HPAA stimulation, when compared to Boer goats (a hardy goat breed) and Merino sheep. 

This review explores the potential of utilizing the HPAA function to improve robustness in sheep. The possibility of simultaneously improving both animal welfare and production with this approach is explored, with some lessons learnt from a divergent breeding programme with South African Merinos. A thorough discussion of HPAA function follows, wherein we explore the potential sites for identifying genetic markers that contribute to variability in HPAA function. The identification of these genetic markers may have potential in marker-assisted selection (MAS) to improve animal welfare and production simultaneously.

## 2. Lessons from the South African Sheep Industry

### 2.1. Current Breeding Strategies in South Africa

The South African small stock industry contributes up to 8% of the total agricultural income from animal products, of which 65.3% is derived from meat (sheep and goats), 30.7% from wool, and 4% from mohair [13]. Although South Africa exports 77% of the produced wool, it imports 28% of its mutton and lamb [14]. Merino and Merino-type breeds constitute >50% of the national sheep flock [5], while Merinos are also the most extensively studied sheep breed worldwide [13,15,16]. The Merino breed had a major impact on the sheep populations of all the major sheep-producing countries in the past century, and Merinos contribute up to a third of all main breed types in some way [15,17].

**Figure 1:**
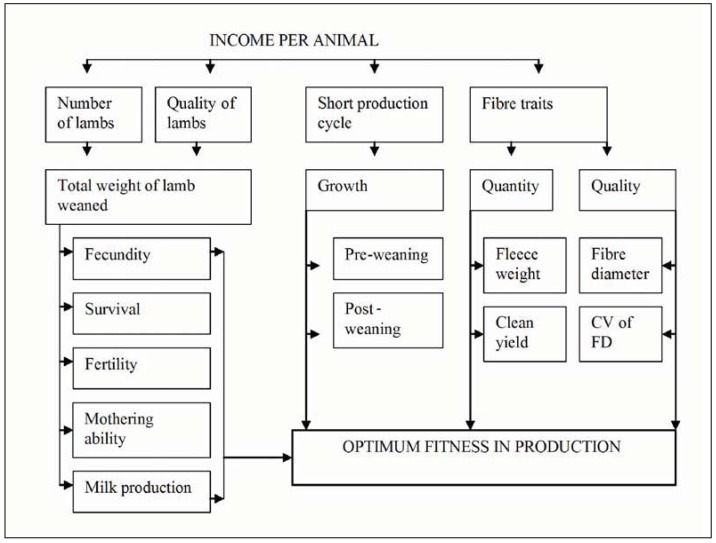
Summary of the strategy proposed by the National Small Stock Improvement Scheme for the genetic improvement of cost per animal. CV: coefficient of variation; FD: fibre diameter. Adapted by S.W.P. Cloete from Olivier [18].

Livestock recording and evaluation programmes, such as the National Small Stock Improvement Scheme in South Africa, have been developed to assist sheep farmers to select genetically superior animals [19,20]. The improved levels of production in such animals are expected to assist in the fight against rising input costs. A diagrammatic summary of this strategy is shown in Figure 1. The strategy proposes the genetic improvement of income per animal by increasing the total weight of lamb weaned, growth rate, as well as quantity and quality of fibre produced [18]. Genetic selection based on these parameters is aimed at improving production traits (e.g., fibre yield, meat quantity) or fitness traits (e.g., lamb survival). Genetic selection is also a preferable alternative to improve fitness and production compared to some husbandry procedures, which increases the cost per animal and may be detrimental to the animals’ welfare (such as mulesing) [15].

### 2.2. Selection Based on Maternal Ranking Values to Rear Multiple Offspring

The Institute for Animal Production of the Western Cape Department of Agriculture, at Elsenburg research farm in South Africa, embarked on a strategic breeding programme in 1986, where selection responses to divergent selection for ewe multiple rearing ability was assessed. The proposal was that selection for this trait would include characteristics for both fitness (increased lamb survival) and “efficiency” of animal production (number of lambs reared per ewe joined). Two distinct Merino lines were established that showed a marked divergent response in overall reproduction, production, and animal welfare. 

The annual rate of genetic improvement in total weight of lamb weaned per lambing opportunity, in the line selected for multiple rearing ability (H-line), was 1.8% of the overall phenotypic mean [21]. A corresponding annual decline of 1.2% of the overall mean was found in the line selected against multiple rearing ability (L-line). These nearly symmetrical responses to genetic selection resulted in an average difference of almost 6 kg (*P* < 0.01) for total weight of lamb weaned (H-line: 23.9 ± 1.2 kg *vs.* L-line: 18.0 ± 1.3 kg) per lambing opportunity between the two lines [21]. Accordingly, the number of H-line lambs born and weaned per ewe was significantly higher (*P* < 0.01) than contemporaries of the L-line, while the H-line ewes seemed to lamb 2.6 days earlier (*P* < 0.01) in the lambing season than L-line ewes [21]. Despite a higher lambing rate, lamb survival was also improved in the H-line compared to the L-line, especially in multiples [22]. These line differences were supported by responses in behaviour conducive to an improved lamb survival rate. H-line ewes displayed improved maternal care compared to L-line ewes [23], where H-line ewes typically experienced shorter births; remained at their birth sites longer; were less likely to desert lambs [22]; groomed lambs immediately after birth; remained with lambs 90–120 min after birth instead of grazing [24]; and were able to reunite with lambs (at 1 day of age) quicker after forced separation, compared to L-line ewes [25]. H-line lambs typically progressed quicker from their first standing after birth to suckling [22], and were more likely to bleat when separated from their mothers [25] than L-line lambs. There were no line differences in the time lapsed from birth to first standing [22], birth weight and birth coat score [26]. This project demonstrated that genetic change in lamb survival would accrue when selection is based on a correlated trait such as maternal ability to rear multiple offspring [27]. These criteria increased both the “efficiency” of production for sheep farmers and the overall fitness of sheep.

The implications of these selection criteria for the H- and L-lines for production traits were subsequently investigated. In terms of wool production, the staple strength of L-line ewes between the ages of 3–6 years were higher than H-line ewes, while the production of clean wool was markedly improved in the L-line across all ages [28]. This was ascribed to the greater metabolic demand of a higher reproduction rate in the H-line, since there was no evidence of genetic divergence for fleece weight in progeny of the two lines [29]. There was no line difference in fibre diameter between the two selection lines [28]. Measurements of live weight and wrinkle score indicated favourable conditions for the H-line, whereas H-line sheep were typically heavier and plainer than L-line sheep [28,30]. Consequently, it was shown that the H-line is markedly more resistant to breech strike [31], since excessive skin folds have conclusively been linked to a higher susceptibility to breech strike [32,33,34] and reduced reproduction potential [35,36,37]. The penalties in wool traits from selection for maternal multiple rearing ability should therefore be balanced against the improvement in income derived from the production of lamb, as well as considering the advantages for animal welfare [31]. A study on the infestation of H- and L-line ewes with sheep lice (*Bovicola ovis*) showed no significant difference in susceptibility to infestation, but produced some evidence that would suggest that H-line sheep could be more tolerant of *B. ovis* than L-line sheep. The selection criteria of the H- and L-lines may well be used to breed a robust and easily managed sheep genotype that might be able to adapt to a wide range of environmental conditions [31].

Further implications for animal welfare in the H- and L-lines were demonstrated by their respective responses to psychological and physiological stress. Stressful behaviour in response to flock isolation was assessed during an arena test, which monitored the movement within the arena (number of lines crossed), distance from human operator (separating the sheep from its flock), number of bleats, number of urinating events and number of defecating events [38]. H-line animals allowed smaller distances between themselves and a human operator situated between them and their flock mates, while the L-line defecated more frequently throughout the stress test [38]. These behavioural responses to psychological stress provided evidence that the H-line was able to adapt to unfamiliar conditions more successfully than the L-line. Furthermore, meat characteristics for these sheep were assessed at time of slaughter [38,39]. It was found that, at the same age and under the same management conditions, the mean slaughter weight, dressing percentage, carcass weight and vivid red meat colour was higher in the H-line than in the L-line (*P* < 0.05) [38]. There was no difference between the two lines in cooking loss, drip loss, and tenderness of meat. The initial pH values, measured in the *Musculus longissimus dorsi*, was lower in the L-line compared to the H-line, which indicated an increased susceptibility of L-line sheep to acute, short-term stress prior to slaughter. Further investigation into the responses of the selection lines to physiological stress revealed that adult H-line rams are able to elicit a higher cortisol response to insulin-induced hypoglycemic stress than L-line rams, with a subsequent quicker blood-glucose recovery to resting conditions [40,41,42]. 

These collective results provided strong evidence that selection for maternal multiple rearing ability resulted in an increased ability to adapt to short-term stress, which seemed to be the result of indirect selection for a more responsive HPAA activity. However, further evidence is still required in future studies to obtain real data on the HPAA function of the H- and L-line sheep (e.g., levels of expression of relevant receptors and enzymes or neural plasticity). Throughout this review, we will discuss the potential sites within the HPAA where these differences between the H- and L-lines are likely to be found. This discussion is based on current observations available from the breeding programme and is likely to be updated as further research results become available. At this stage, however, it is clear that the adopted selection strategy seems to have elicited favourable responses in both production and animal welfare.

## 3. Robustness as Breeding Goal: Utilization of HPAA Function

Not all breeders have the same production-related objectives, but for any genetic selection programme to be efficient, one would ideally need to incorporate quantitative traits for both robustness- and production-related traits as selection criteria to reach sustainable breeding goals. Most quantitative traits are determined by an intricate network of interacting loci and environmental factors [43]. In the best of circumstances, the genetic variation of a quantitative trait is determined by a small number of major loci with moderate to large effects, together with a large number of minor loci with small effects, known as the oligogenic model [44,45]. The identification of these loci, or markers in linkage disequilibrium with it (called quantitative trait loci or QTLs), in the genome is fundamentally important for agriculture in terms of MAS or marker-assisted management [46,47,48]. These methods aid the speed and accuracy of estimating breeding values in genetic selection programmes or to adapt management practices to better match the genotypes of livestock (e.g., feeding, pre-slaughter procedures and drug therapy). 

The foregoing section on H- and L-line sheep illustrated that it is possible to make genetic progress in HPAA function. Large individual variation in the HPAA function has previously been described that have important physiopathological consequences [48]. Variation within the responsiveness of the HPAA to various types of stressors, as well as moderate-to-high heritability estimates thereof, suggests that there are important genetic factors that can influence this phenotype [49,50,51,52]. Many of these genetic factors have been identified, such as polymorphisms in specific genes [1,53,54,55,56], as well as tissue-specific epigenetic variants that were established during early-life development [57,58]. 

The glucocorticoids (cortisol or corticosterone) released by the adrenal gland on HPAA stimulation, exert a wide range of effects, including effects on metabolism, inflammatory processes and the immune system. Individual variation has also been reported in the production of these glucocorticoids, their bioavailability, as well as receptor and post-receptor mechanisms, which are areas that may be targeted during selection. The integration of these sources of genetic variability allows for the development of a model for MAS to improve animal robustness without the negative side effects on production [1]. Alternatively, various stress tests, such as a flock-isolation test, may be used to identify individuals with superior HPAA function for breeding purposes. Some agricultural studies have attempted to identify phenotypic traits (such as litter size) that are associated with glucocorticoid release from the adrenal gland [59,60], but these traits are different among species and breeds. Some of these differences may be attributed to the inter- and intra-species differences in glucocorticoid production within the adrenal gland.

The review by Mormède *et al.* [61] illustrated the utilization of the HPAA function to assess animal welfare in farm animals. In a follow-up review, Mormède *et al*. [1] demonstrated that the variability within the HPAA function could be utilized to identify loci that affect robustness in farm animals that may prove useful in MAS. It was clear that the assessment and interpretation of the glucocorticoid response on stimulation of the HPAA is not always straightforward, since the effects of cortisol seemed to have both positive and negative effects on production-related traits, with specific reference to the pig industry. This review expands on the latter contention, using observations from the South African sheep industry, by suggesting that an effective acute stress response is beneficial for animal welfare and production, whereas inefficient stress responses or prolonged exposure to stress is detrimental. For this reason, a discussion about stress and its relevance for the sheep industry is necessary. 

### 3.1. Introduction to Stress

The importance of the capacity of an animal to cope with stressors is perhaps better understood by defining stress and its consequences. Selye [62] defined “stress” as the disease of adaptation, where the mechanisms to cope with stressors become overextended and eventually break down. Ewbank [63] extended this idea and define three phases that are seen as a continuum of responses to a stressor: stress, overstress, and distress. Stress is when an animal copes with a stressor within its capacity at an adaptive and harmless level. Overstress is when the coping mechanism is extended, but still remains sufficient to counteract the stressor. Distress, however, is when a stressor stretches the coping mechanism beyond its limits to the point where the response is non-adaptive and results in damage to the animal’s health, identified as the disease of adaptation. 

The adaptive mechanisms for coping with stress lie within the nervous system, immune system, endocrine system and the interregulation between these systems [61]. When an animal encounters a stressor two stress responses are activated, namely the fast fight-or-flight response via the sympathetic nervous system (within seconds) and the slower glucocorticoid response (within minutes) via the HPAA [64]. The fight-or-flight response results in the release of epinephrine and norepinephrine from the adrenal medulla, which enables the animal to respond quickly to the stressor by increasing heart rate and blood pressure, while mobilizing energy sources to the central nervous system and somatic muscle. The slower glucocorticoid response from the adrenal cortex follows the fight-or-flight response via the hormonal cascade of the HPAA. The glucocorticoids also mobilize energy sources and their various mechanisms of action will also be discussed in greater detail in this review. The glucocorticoids exert a negative feedback effect on the HPAA and the production of glucocorticoids is suppressed as the stressor decreases or cease. 

Together the glucocorticoid and fight-or-flight responses constitute the acute stress response that serves to functionally divert physiological and behavioural processes to aid in the immediate survival requirements of the animal, a condition called the “emergency life-history stage” [65]. At this stage, the other normal life-history functions (e.g., reproduction, growth, and immunity) are suspended so that physiological and behavioural functions are focused on coping with the immediate danger the stressor presents. This stress response is crucial for the animal to survive an immediate stressor (such as sight of a predator and handling during husbandry procedures). For instance, animals that were able to mount a higher HPAA response showed increased tolerance to heat stress [66,67] or an increased resistance to bacteria and parasites [2,52,68].

However, if a series of stressors initiate multiple consecutive stress responses or if the animal is continually exposed to a single stressor for a long period of time (chronic stress or inability to cope with stress), the continuous activation of this stress response becomes detrimental to the health of the animal [69]. For example, an environmental factor that continually stimulates the HPAA for numerous days will lead to an increase in cortisol secretion from the sheep adrenal cortex over an extended period of time. These elevated cortisol levels over a prolonged period of time will inhibit inflammatory processes to the point where it will eventually increase the animal’s susceptibility to pathogens [15,70].

### 3.2. Stress in Sheep

Stress in farm animals is more common than one may initially consider and is unavoidable in farming practises. This source of stress may be due to deviations in physiological homeostasis (e.g., drop in blood-glucose), while it could also be of a real (e.g., environmental conditions or sight of predator) or perceived (e.g., emotional stress such as flock-isolation) nature. Sheep are frequently subjected to routine handling procedures and some differences in ease of handling between breeds have been reported, where Merinos have been found to be one of the easiest to handle [15]. Kilgour [71] suggested that three basic behaviours of sheep should be recognized for successful sheep handling: (1) their strong flight reaction; (2) the prevailing role of vision in social organization; and (3) their flocking-follower behaviour. It is thus understandable why sheep avoid isolation from the flock, which results in unpredictable fearful behaviour and sometimes leads to injury during routine handling procedures [72,73]. 

Lynch *et al*. [15] report that the separation of sheep from their flock, and the anxiety it causes, is likely to be the predominant source of suffering and is considered a potent stimulus of the HPAA. Various studies have also shown that some husbandry practices, such as shearing, crutching, drafting and transport, resulted in increased plasma cortisol levels [72,73,74,75]. Cold stress and starvation are two other important factors that stimulate the HPAA. Starvation and cold exposure are two of the four main, and often interrelated, factors associated with most lamb deaths, along with difficult parturition and relatively low birth weight, according to the review of Alexander [76]. 

Murphy *et al*. [77] and Murphy [78] have been able to correlate lamb survival with ewe temperament. These authors based their findings on Merino ewes selected for either high (“nervous”) or low (“calm”) reactivity to humans and flock-isolation. The mortality rate of lambs born to “calm” ewes was half that of “nervous” ewes. The authors proposed that the higher lamb survival could be ascribed to the display of behaviour conducive to superior maternal care, in terms of grooming and bleating frequency, compared to the “nervous” ewes [77,78]. Furthermore, the degree to which maternal behaviour is displayed has been correlated with the concentrations of cortisol, progesterone and estradiol during the peripartum period [79,80]. However, Bickell *et al*. [81] showed that the concentrations of progesterone and estradiol were similar in the two temperament lines from 4 days prior and 24 hours *post* parturition, which indicated that it was unlikely that these hormones contribute to the displayed maternal behaviour. The latter study also failed to support a hypothesis that “calm” ewes and lambs coped better with the situation than their “nervous” counterparts when they were subjected to a test involving a novel distraction during the early postnatal phase.

As mentioned previously, selection for maternal multiple rearing ability (H-line) resulted in “calmer” sheep [38], where ewes displayed superior maternal care [22,24,25] and there was a subsequent increase in lamb survival [27]. Although the H-line was able to elicit a higher cortisol response to physiological stress than the L-line, it is unknown at this stage whether this difference in cortisol production is present during the peripartum period and responsible for mediating some of the behaviours associated with superior maternal care.

The collective observations from these studies demonstrate the complex relationship between stress, production, and reproduction. Incidentally, these results cannot be accepted as universal. A similar study involving an Australian Merino line selected for reproduction (the Fertility flock), and a randomly bred control line, failed to show conclusive evidence in behaviour in favour of ewes from the Fertility flock during contrived situations [82]. The phenomenon that stress is accompanied by a reduced reproduction is, however, well known and was observed from 1946 by Selye [62]. This phenomenon is attributed to the preservation of adrenal cortex activity at the expense of gonadal activity in the life emergency-history stage [83]. Stress-related hormones, such as corticotrophin-releasing hormone (CRH), proopiomelanocortin-derived peptides (e.g., adrenocorticotrophic hormone and β-endorphins) and adrenocorticosteroids (e.g., cortisol), can influence sexual function on all three levels of the hypothalamic-pituitary-gonadal axis (HPGA), namely the brain (inhibits secretion of gonadotropin releasing hormone), the anterior pituitary (CRH inhibits luteinizing hormone secretion), and the gonads (alters stimulatory effect of gonadotropins on sex steroid secretion) [83]. It is therefore not so surprising that “calmer” ewes have a superior reproductive rate than their “nervous” contemporaries.

### 3.3. The Hypothalamic-Pituitary-Adrenal Axis

The HPAA mediates stress responses in combination with the autonomic nervous system and behavioural adaptation [84]. The hypothalamus receives neuronal input from various internal and external stimuli and conveys this signal to the anterior pituitary via CRH and arginine vasopressin (AVP). The synergistic action of CRH and AVP stimulate the secretion of ACTH from the anterior pituitary gland. ACTH in turn stimulates the release of glucocorticoids from the adrenal cortex, of which cortisol is the primary glucocorticoid in sheep. The main active hormone in the HPAA response for most mammals and fish is cortisol, whereas corticosterone is the only active glucocorticoid for most rodents and birds [61]. The measurement of circulating cortisol (or corticosterone for birds and rodents) is universally accepted as the golden standard of evaluating stress and welfare in animals, since it reflects the responsiveness of the HPAA to a stressor [1]. However, various biological samples have been used successfully to measure HPAA function, such as saliva, urine, faeces and milk [85]. However, in these alternative samples, a change in cortisol concentration is reflected after a delayed period of time (sometimes hours to days) and the cortisol concentrations in these substances are also typically lower.

Various differences in HPAA activity have been found across species, breeds and individuals, which reflects the contribution of genetic factors and environmental influences to the large variability within the system [60,86]. This aspect of HPAA function makes genetic selection for superior HPAA activity a promising tool in animal breeding [1]. Some of the other sources of variation arise from the pulsatile, diurnal and seasonal rhythms in secretion of adrenocorticosteroids, which is also influenced by physiological state, age, sex, feed intake and environmental factors such as temperature and humidity [61]. 

In the following sections, we will discuss the potential contributions of the hypothalamus, pituitary, adrenal cortex and mechanism of action for glucocorticoids, respectively, towards the genetic variability within the HPAA function.

#### 3.3.1. The Hypothalamus

One of the most pronounced features of the HPAA is its nyctohemeral cycle, which is controlled by neuronal pacemakers in the paraventricular nucleus of the hypothalamus [87]. This neuronal activity under resting conditions is not markedly altered by glucocorticoids. However, the effects of glucocorticoids on neuronal activity become apparent once the neurons are activated by neurotransmitter input that exceeds resting conditions (e.g., during signals of stress) [84].

The hypothalamus controls the release of ACTH from the anterior pituitary gland by a neuronal structure known as the paraventricular nucleus (PVN) [84]. The parvicellular subdivisions of the PVN include specialized neurons that synthesize CRH and AVP, which is released in the capillary bed of the median eminence where it reaches the pituitary via the hypothalamic-pituitary portal vessels [88]. The axons of the magnocellular part of the PVN extend into the posterior pituitary to release AVP and oxytocin [84,89]. It has been proposed that AVP maintains HPAA activity during prolonged stimulation, whereas CRH seems to be mainly active during the acute stress response [84,90,91]. The PVN receives numerous inputs from the brain stem (neural inputs from the periphery), hypothalamic nuclei (metabolic and nyctohemeral inputs), limbic system (in relation to emotional state) and subfornical organ system (monitors blood plasma composition) [84]. The complexity of the various afferent and efferent pathways connected to the PVN explains why the HPAA is sensitive to a wide range of external and internal stimuli to assess the homeostatic state of the sentient organism. This enables the hypothalamus to communicate a state of stress (or normality) to the anterior and posterior pituitary as to which hormones to release for the maintenance of homeostasis.

Da Costa *et al*. [73] studied emotional stress and its effects on the HPAA of sheep. These researchers subjected sheep to flock-isolation and found that showing facial pictures of familiar sheep, compared to pictures of goats and inverted triangles (control group), reduced the stress responses of these sheep, in terms of behavioural (reduced activity and protest vocalizations), autonomic (decreased heart rate) and endocrine (decreased cortisol and adrenaline) indices of stress. Their mRNA expression of activity-dependent genes (c-*fos* and *zif*/268) was also found to be reduced in the PVN and the brain regions associated with fear (central and lateral amygdale), while their expression was increased in the brain regions dedicated to emotional control (orbitofrontal and cingluate cortex) and for processing faces (temporal and medial frontal cortices and basolateral amygdala). This study indicated the role of the PVN to translate signals of emotional stress via the HPAA to increase or decrease cortisol levels accordingly [73]. Furthermore, the emotional reactivity (temperament) of sheep has been successfully used as selection criterion in breeding programmes to improve reproductive biology [92]. Selection for “calm” ewes increased lamb survival and maternal behaviour [77]. The activity of the PVN has been shown to affect mother-young relationships of sheep, and the expression of c-*fos* in the PVN has been used as a marker for neuronal activity that correlated with the onset of maternal behaviour after parturition [93,94]. 

It might be plausible for the H- and L-line ewes, that were previously mentioned, to have different PVN activities (or other neurological differences), since they displayed distinct maternal behaviours after parturition [21,22,23,24,25], as well as distinct behavioural responses to flock-isolation stress (arena test) [38]. It is noteworthy that in the arena test these sheep had visual contact with other members of their flock while being separated by a fence and a human, which implies that signals of fear, facial recognition and emotional stress were most likely translated to the PVN like Da Costa *et al*. [73] demonstrated. These observations supply evidence that investigations into the function of the hypothalamus for the H- and L-lines might aid in the identification of genetic factors that are responsible for the differences in maternal care and behavioural stress responses observed for these selection lines. These investigations may for instance be directed at determining the expression level of indicator genes or receptors, as well as neural plasticity.

#### 3.3.2. The Pituitary

The hypothalamus is connected to the posterior pituitary by axons that extend down from the paraventricular and supraotic nuclei through the infundibulum [95]. These axons secrete AVP and oxytocin, as previously mentioned, via exocytosis into the posterior pituitary capillaries, which drain directly into the main blood circulation [84,89]. AVP facilitates the re-uptake of water in the kidney by increasing the permeability of the collecting ducts. Oxytocin increases contraction of smooth muscles in the mammary glands and uterus [95]. 

The hypothalamus is connected to the anterior pituitary by a special vascular system, the hypothalamic-pituitary portal system, which ensures that blood flows directly from the hypothalamus to the anterior pituitary [95]. 

In the anterior pituitary, CRH binds to corticotrophs, specialized secretory cells, to release ACTH [95]. In addition to corticotrophs, the anterior pituitary also consists of four other types of secretory cells that are responsible for the production and secretion of different trophic hormones. Each secretory cell type responds to a specific hypophysiotrophic hormone secreted by various neurons in the hypothalamus into the median eminence. The secretory cells in the anterior pituitary consist of 20% corticotrophs (secretes ACTH in response to CRH); 50% somatotrophs (secretes growth hormone in response to growth hormone-releasing hormone and growth hormone-inhibiting hormone); 20% mammotrophs (secretes prolactin and regulated by prolactin-inhibiting hormone); 5% thyrotrophs (secretes thyroid-stimulating hormone in response to thyrotrophin-releasing hormone) [95]; and 5% gonadotrophs (secrete follicle-stimulating hormone and luteinizing hormone in response to gonadotropin-releasing hormone). Various studies have shown that CRH inhibits the stimulation of gonadotrophs to release luteinizing hormone (for review see Rivier and Rivest [83]). It is therefore not surprising that stress inhibits the reproductive endocrine axis in farm animals [96].

The binding of CRH to the CRH receptor of the corticotrophs activates adenylate cyclase and the accumulation of cyclic adenosine monophospate (cAMP) subsequently activates protein kinase A [97]. This stimulation of adenylate cyclase by CRH is regulated by divalent ions and guanidine nucleotides, a common phenomenon observed for receptors coupled to adenylate cyclase [98]. The synergistic action of CRH and AVP is important in the physiological control of ACTH secretion. AVP requires the presence of CRH to exert its full effect. AVP has a weak ACTH-releasing activity *in vitro* for most species, and instead potentiates both CRH-stimulated ACTH release as well as CRH-induced accumulation of cAMP. AVP act via a V1-like receptor that results in the stimulation of phosphatidylinositol hydrolysis and intracellular calcium ion fluxes that subsequently activates protein kinase C. Studies on sheep have shown that the relative potencies of CRH and AVP are reversed, where CRH has a weak ACTH-releasing activity, but can potentiate the effects of AVP on ACTH release [99,100,101]. Furthermore, AVP and ACTH secretion is stimulated by interleukin-6 [102,103].

ACTH is a polypeptide that is produced by the cleavage of the larger polypeptide proopiomelanocortin (POMC) [104]. Cleavage of POMC also yields endorphins (endogenous opioids) and lipotrophins (implicated in lipid metabolism) that can be secreted together with ACTH from corticotrophs in small quantities. ACTH enters the main blood circulation via the anterior pituitary capillaries and reaches the adrenal gland where it stimulates the secretion of glucocorticoids, primarily cortisol, from the adrenal cortex of sheep. ACTH may stimulate the secretion of mineralocorticoids and androgen precursors in other species, but it depends on the steroid biosynthesis pathway and physiological requirements of that particular species [95].

It has been suggested that there is a maturational change in the heterogeneity for populations of foetal corticotrophic cells in late gestation [105]. The one type of corticotrophic cells are CRH-responsive and primarily secrete ACTH, while they are also sensitive to glucocorticoid-related inhibition [106]. The other type of corticotrophic cells are responsive to AVP only and contribute relatively more to the secretion of ACTH-precursors than ACTH itself, while they are not sensitive to glucocorticoid-related inhibition [106]. This heterogeneity in foetal corticotrophic cells might aid in the continual activation of the foetal HPAA in late gestation that result in the typical cortisol surge (and elevated ACTH concentrations) in the final 10–15 days preceding parturition [107], which is presumed to be dependent on the secretion of CRH, as well as AVP, from the neurons of the foetal PVN [105]. This cortisol surge therefore originates primarily from the foetal adrenal cortex (*vs.* maternal adrenal cortex) and is crucial in the final maturation of foetal vital organs, as well as the onset of the cascade of events that initiate parturition [105]. As previously mentioned, the H-line typically experience births 2.6 days earlier in the lambing season than the L-line [21], while adult H-line rams had superior HPAA responsiveness in terms of being able to elicit a higher cortisol response to physiological stress [40,41,42]. It is feasible that selection resulted in differences between the H- and L-lines in their foetal maturational changes in the heterogeneity of corticotrophic cell populations, or other changes in anterior pituitary function (such as sub-cellular signalling responses to CRH; post-translational processing of POMC; or response to glucocorticoid feedback). This would allow for an earlier or higher cortisol surge in late gestation for the H-line compared to the L-line and could contribute to the shorter gestation periods for the H-line. However, the heterogeneity of corticotrophic cells in the H- and L-line foetus, or assessment of other anterior pituitary functions, has not been investigated to date and these remarks are purely speculation at this stage, but provide evidence for a site where potential genetic markers may be identified.

#### 3.3.3. The Adrenal Gland

In the adrenal cortex ACTH binds to the ACTH receptor on the outside of the cell membrane. This transmembrane ACTH receptor is associated with a signal transducing G-protein, G_s_, which is located on the inside of the cell membrane [95]. Activation of G_s_ increases cytosolic cAMP that binds to, and activates, cAMP-dependent protein kinases. The activated protein kinases can alter the catalytic activity of numerous enzymes by means of phosphorylation, including ribosomal phosphorylation, at specific serine and threonine residues. These responses lead to an increase in secretion of glucocorticoids [108]. 

Glucocorticoids, like all other steroid hormones, cannot be stored in adrenocortical cells, and subsequently their supply is dependent on *de novo* synthesis from the common precursor, cholesterol [108]. Therefore, much of the control over the glucocorticoid response resides in the activity of the steroidogenic enzymes that facilitate their biosynthesis. 

Various factors are involved in the supply, transport and storage of cholesterol in adrenocortical cells. The major source of cholesterol in sheep originates from low-density lipoproteins (LDL) in plasma, which is derived primarily from dietary cholesterol [109]. Access to LDL is accomplished by the high degree to which adrenal tissue is vascularized [110]. Adrenocortical cells can acquire circulating LDL via LDL-mediated endocytosis [111]. The cholesterol esters are subsequently hydrolysed within the endosome by lyposomal acid lipase to release cholesterol [112].

Adrenocortical cells are also able to synthesize cholesterol *de novo* from acetate in the endoplasmic reticulum [113]. Intracellular cholesterol, irrespective of origin, can be esterified with fatty acids in the endoplasmic reticulum, where cholesterol esters accumulate and bud off as lipid droplets [108]. This esterification of cholesterol is catalysed by acyl-coenzyme A: cholesterol acyltransferase (ACAT). Cholesterol esters from lipid droplets can be accessed and hydrolysed in turn by cholesterol ester hydrolase and neutral cholesterol ester hydrolase (HSL), but the relative contribution of these two enzymes is not known [114]. The intracellular fate of cholesterol is largely regulated by sterole response element binding proteins (SREBPs) [115]. These proteins belong to a group of transcription factors that generally regulate genes involved in the biosynthesis of cholesterol and fatty acids. The rate-limiting enzyme in cholesterol synthesis, known as 3-hydroxy-3-methylglutaryl co-enzyme A reductase, is activated by ACTH, while it is suppressed by adequate LDL concentrations. Cellular cholesterol is increased by ACTH (within three minutes after ACTH treatment), which also stimulates HSL, LDL uptake and transcription of LDL receptors, whereas it inhibits ACAT [108].

Cholesterol is virtually insoluble in aqueous solutions and is therefore transported through the cytoplasm by binding to proteins [108]. Once cholesterol reaches the outer mitochondrial membrane, it is transported across to the inner mitochondrial membrane by the steroid acute regulatory protein (StAR) [116,117]. When cholesterol reaches the inner mitochondrial membrane, its conversion to pregnenolone is facilitated by cytochrome P450 cholesterol side-chain cleavage (CYP11A1). This is considered the first committing and rate-limiting step in adrenal steroidogenesis and its regulation by multiple mechanisms makes it a finely tuned control point for quantitative regulation of steroid hormone biosynthesis [108]. The type of steroid hormone to be produced (qualitative regulation) is determined by the mechanism of action of the remaining steroidogenic enzymes in the pathway and their cofactors (e.g., NADH, NADPH and cytochrome *b*_5_). 

These enzymes are selectively expressed in the morphologically and functionally distinct zones of the adrenal cortex, namely the *zona glomerulosa* (outer most layer, primarily for mineralocorticoid synthesis), *zona fasciculata* (centre layer, primarily for glucocorticoid synthesis) and *zona reticularis* (innermost, primarily for androgen synthesis). These steroidogenic enzymes generally belong to one of two major classes of proteins, known as the heme-containing cytochrome P450 proteins and the hydroxysteroid dehydrogenases. These proteins are bound to the membranes of either the mitochondrial or endoplasmic reticulum. The adrenal steroidogenesis pathway is therefore stretched across different morphological zones and intracellular compartments, while some steroidogenic enzymes catalyse more than one reaction. The complexity of the adrenal steroidogenesis pathway emphasizes the requirement for precise control over the qualitative regulation of steroid biosynthesis to meet physiological demands [108].

Once cholesterol is converted to pregnenolone by CYP11A1 in the mitochondria, pregnenolone can relocate to the endoplasmic reticulum, where it serves as substrate for either CYP17 or 3β-hydroxysteroid dehydrogenase/∆^5^→∆^4^ isomerase (3βHSD) [108]. Pregnenolone is hydroxylated at C-17 by CYP17 to yield 17-hydroxypregnenolone, which in turn acts as yet another substrate for CYP17. In this step, the bond-cleavage between C-17 and C-20 of 17-hydroxypregnenolone results in the formation of dehydroepiandrosterone (DHEA). The C-3 dehydrogenation of the ∆^5^ steroids, namely pregnenolone, 17-hydroxypregnenolone and DHEA, by 3βHSD converts these metabolites to their ∆^4^ isoforms, namely progesterone, 17-hydroxyprogesterone and androstenedione, respectively. Furthermore, CYP17 also mediates the hydroxylation of progesterone at C-17 to yield 17-hydroxyprogesterone, as well as the bond-cleavage between C-17 and C-20 of 17-hydroxyprogesterone to yield androstenedione. Sheep CYP17 has been reported to hydroxylate C-16 of progesterone [56], which is an activity of CYP17 that has also been reported for the human, baboon and Angora goat [118]. Progesterone and 17-hydroxyprogesterone then acts as substrates for cytochrome P450 21-hydroxylase (CYP21) in the endoplasmic reticulum, which hydroxylates these steroid metabolites at C-21 to respectively yield deoxycorticosterone and 11-deoxycortisol. A single mitochondrial enzyme, namely cytochrome P450 11β-hydroxylase (CYP11B), mediates the 11-hydroxylation of 11-deoxycortisol to cortisol in sheep, as well as all three steps required for the synthesis of aldosterone from deoxycorticosterone, namely the 11-hydroxylase (yields corticosterone), 18-hydroxylase (yields 18-hydroxycorticosterone) and 18-methyl oxidase (yields aldosterone) activities [119]. In humans, these three steps are mediated by more than one enzyme [108]. Kinetic studies have shown that CYP11B binds preferentially to deoxycorticosterone compared to corticosterone and 18-hydroxycorticosterone, and that these two latter intermediates are not released from the active site of the enzyme [119,120]. Although CYP11B is expressed in all three zones of the adrenal cortex in sheep, cattle and pig species, the synthesis of aldosterone is only observed in the zona glomerulosa and the reason remains unknown [119,121].

A study by Hough *et al*. [56] clearly demonstrates the potential of using the variability within adrenal steroidogenesis to improve the responsiveness of the HPAA. Two isoforms for ovine CYP17 were identified and *in vitro* characterization of their activities predicted that the Wild Type 1 isoform (WT1) would be beneficial for cortisol production, since it is able to produce more cortisol-precursors than the Wild Type 2 (WT2) isoform. When adult South African Merino rams were subjected to insulin-induced hypoglycaemic stress, the homozygous *WT1/WT1* rams elicited a markedly higher and more rapid (*P* < 0.05) cortisol response than heterozygous *WT1/WT2* rams (peak cortisol concentrations: 120.9 ± 14.6 mmol/L *vs.* 92.1 ± 8.7 mmol/L). The effect of the CYP17 genotype on behavioural stress responses to flock-isolation was also investigated in H- and L-line sheep (n = 400) [42]. These results indicated that the CYP17 genotype is the major factor, rather than the selection line, that affects behavioural responses in the arena test, which is most likely mediated via the rapid cortisol response associated with the WT1 isoform [42]. Homozygous *WT1/WT1* sheep were more likely to allow smaller distances between themselves and a human (*P* < 0.05), whereas they had fewer protest vocalisations (*P* < 0.01), but urinated more frequently (*P* < 0.01). It is therefore reasonable to consider the two SNPs located within the CYP17 genotype as potential genetic markers of robustness. However, it needs to be stated that MAS in an already robust genotype, such as the H-line, is less likely to result in significant improvement in robustness than in a less robust genotype, like the L-line [42].

It is not only the variability within the activity of steroidogenic enzymes that may influence the cortisol response from the adrenal cortex, but also the regulatory mechanisms that influence the adrenal cortisol response to ACTH stimulation in a complex manner. ACTH stimulation regulates adrenal steroidogenesis at three levels [63,108]. First, the acute regulatory response occurs within minutes and mostly respond in a way that increase the availability of cholesterol and its delivery to the inner mitochondrial membrane for conversion to pregnenolone by CYP11A1. The transport of cholesterol from the outer to inner mitochondrial membrane by StAR is sometimes considered to be the main regulator of the acute ACTH response [108]. However, the exclusive regulation of cholesterol availability in the acute response would result in a general increase of all the steroid hormones and it is well known that ACTH stimulation specifically increases glucocorticoid output in adrenocortical cells. Therefore, in the acute response, the basal expression of CYP11A1, CYP17, CYP21 and CYP11B in the *zona fasciculata* needs to be in favour of glucocorticoid production, or other non-transcriptional regulating factors must be involved in eliciting a glucocorticoid specific response. These factors include the increase in blood flow to the adrenal gland [122,123,124]; serine/threonine phosphorylation of CYP17 to decrease 17,20-lyase activity relative to 17α-hydroxylase activity [125]; and alterations in cytoskeletal structure to improve interorganelle substrate delivery between the mitochondria and endoplasmic reticulum [126,127,128,129,130,131,132,133,134,135,136,137,138].

Secondly, ACTH acts over hours to days via cAMP, whereas angiotensin II act via the calcium/calmodulin pathway, to increase the transcription of steroidogenic enzymes and their cofactors that favours cortisol production [139]. Various mechanisms are in place for the expressional upregulation of these enzymes, but these mechanisms are not the same for each steroidogenic enzyme. The expressional upregulation in response to ACTH stimulation is mostly mediated by activated protein kinases, such as ribosomal phosphorylation, or by cAMP that can act directly via the cAMP response element/cAMP response element binding protein (CRE/CREB) system [139]. Numerous studies have demonstrated the time-dependent increase in expression of steroidogenic enzymes hours after ACTH or cAMP stimulation. For example, Kempna *et al*. [125] observed an increase in CYP17 mRNA in H295R cells 24 hours after cAMP stimulation, but no significant change within the first three hours. Generally the increases in mRNA expression are measured 24 hours after adding stimulation or inhibition agents to the experimental cells [108,139,140,141].

The expressional regulation of steroidogenic enzymes by ACTH is also important for increases in cortisol that is required for the onset of parturition. The increase of cortisol concentrations with the concomitant increase in ACTH in the last 10 to 15 days of gestation is well known for sheep [107]. It has been demonstrated that the expression of CYP11A1, CYP17 and CYP21 is increased 2 to 3-fold in the foetal adrenal and is essential for the increase in adrenal steroidogenesis that precedes parturition [105,142]. 

Thirdly, the long-term exposure to ACTH over weeks to months promotes adrenal growth that results in adrenal cell hypertrophy and hyperplasia. This process is facilitated by intra-adrenal interactions between adrenocortical cells, adrenomedullary cells, nerve fibres and immune cells via their secretory products (e.g., cytokines, growth factors and neurotransmitters). The interaction between these cells allows for complex regulatory circuits, however, further study is still required to elucidate how the secretory products of the adrenomedullary cells are involved in the regulation of adrenocortical activity [122]. In summary, various adrenomedullary secretions, such as catecholamines and a whole series of neurotransmitters, may interact with adrenocortical cells by addition, potentiation or antagonism of their effects. In return, the secretory products of the adrenal cortex, namely steroid hormones and cytokines, influence the expression of proteins, catecholamines and neuropeptides in adrenomedullary cells. There is increasing evidence that the colocalization of medullary and cortical cells is a prerequisite for paracrine interactions within the adrenal gland. Gap junctions have been suggested to play a more important role in communication between these cell types than previously thought and the number of gap junctions increases rapidly with ACTH stimulation. [122,143]. 

Cytokines directly influence adrenocortical function, and are derived from either adrenal cells themselves (primarily cortical cells) or from immune cells that regularly infiltrate the adrenal gland. The localization (adrenal zona) of cytokine producing cells, as well as the type of cytokine produced, varies across species [122]. Generally cytokines like interleukin-1 (IL-1), IL-2 and IL-6 stimulate steroidogenesis (production of glucocorticoids with anti-inflammatory actions) [52,144,145,146], while tumor necrosis factor-α (TNFα) and interferon-γ exert a regulatory influence on adrenal growth. Both the immune system and endocrine system play a crucial role, and interact at different levels, in the adaptive HPAA response to deviations in homeostasis (from stress or disease). It has been suggested that the acute steroidogenic response is regulated at the level of the hypothalamus, while long-term regulation is mediated at the level of the adrenal by the locally produced cytokines, IL-1, IL-6 and TNFα [147].

Furthermore, adrenal cells produce growth factors that locally mediate the development and maintenance of the adrenal cortex [122]. These growth factors include transforming growth factor-β (TGFβ), insulin-like growth factors (IGFs) and β-fibroblast growth factors (βFGF), that mediate a variety of stimulatory and inhibitory effects on the growth and differentiation of the adrenal. These actions of the growth factors may well be the mechanism by which systemic factors, like ACTH, mediate their growth-regulating effects and contribute to their acute and chronic effects on steroidogenesis.

**Figure 2:**
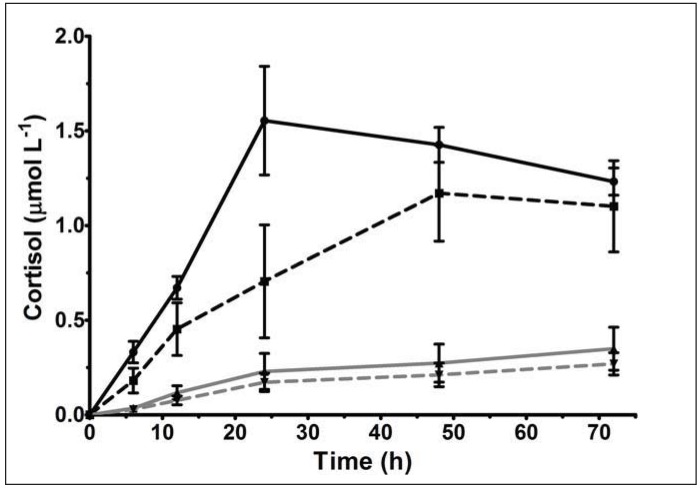
Comparison of cortisol production in adult sheep adrenocortical cells incubated for 72 hours with 100 µM pregnenolone and 1 µM ACTH. Results are expressed as mean ± SEM of triplicate measurements for the adrenocortical cells (primary cultures) prepared from adult rams, representative of each selection line (H- *vs.* L-line) × CYP17 genotype group (homozygous *WT1/WT1 vs.* heterozygous *WT1/WT2*) (n = 2 animals of each group). The H-line is represented by a black line, the L-line represented by a grey line, whereas homozygous *WT1/WT1* groups are represented by a solid line, and heterozygous *WT1/WT2* groups by a broken line. Adapted from Hough [42].

The genetic variability in adrenal function was obvious in a study by Hough [42], where primary cultures were prepared from the adrenal glands of H- and L-line Merino rams (Figure 2). Comparisons of adrenal steroidogenesis under unstimulated and ACTH-stimulated conditions over a period of 72 hours clearly indicated that the adrenocortical cells of H-line sheep produced significantly more (~4-fold) cortisol than L-line sheep throughout the experiment. This indicated that a great degree of variability in the HPAA function of the H- and L-lines resided within the adrenal function. The addition of cholera toxin or forskolin (mimics ACTH-mediated intracellular signalling pathway) instead of ACTH to these adrenocortical cell cultures showed no significant difference between the glucocorticoid responses of the H- and L-line. This observation indicated that the difference in glucocorticoid response between the H- and L-line is unlikely to reside within the ACTH-stimulated intracellular signalling pathway. 

The foregoing discussion highlighted various factors that may potentially contribute to this observed difference in adrenal function of the H- and L-lines. The results of Hough [42] demonstrated the potential to make genetic progress for adrenal function in selection programmes, especially when considering the probable large variability within the system.

#### 3.3.4. Extended Glucocorticoid Metabolism

Before discussing the mechanism of action of cortisol, it is first necessary to explore an extension of glucocorticoid metabolism, which does not necessarily occur in the adrenal gland. These additional steps, mediated by the 11β-hydroxysteroid dehydrogenase (11βHSD) enzymes, play an important role in the metabolism of cortisol and corticosterone, but are still relatively understudied in stress-related assessments. These 11βHSD enzymes mediate the interconversion between the active hormones, cortisol and corticosterone, and their inactive 11-oxo-derivatives, known as cortisone and 11-dehydrocorticosterone, respectively. Sheep 11βHSD types 1 (11βHSD1) and 2 (11βHSD2) have been cloned and characterized [148,149]. The simultaneous assessment of all these glucocorticoids may be more informative than the measurement of cortisol alone (e.g., cortisol:corticosterone ratio). 

11βHSD1 is expressed in numerous tissues that are glucocorticoid responsive (e.g., pituitary gland, brain, lung, bone and eye), but it is most abundant in the adipose tissues and the liver [108]. 11βHSD1 predominantly functions as an oxoreductase to convert cortisone (or 11-dehydrocorticosterone) to cortisol (or corticosterone) using NADPH as cofactor, but also mediates the oxidation of cortisol (or corticosterone) to cortisone (or 11-dehydrocorticosterone) using NADP^+^ as cofactor. The interconversion is dependent on the availability of the cofactor, but can only function with high concentrations (micromolar) of glucocorticoids [108,150]. 

The 11βHSD2 enzyme is predominantly found in target tissues of mineralocorticoids, such as the kidney, brain, colon, testis and placenta [148]. This isoform can only mediate the oxidation of cortisol (or corticosterone) to cortisone (or 11-dehydrocorticosterone) with NAD^+^ as cofactor and functions with low concentrations of steroid (nanomolar). In these tissues 11βHSD2 “defends” the mineralocorticoid receptor from excess glucocorticoids (able to bind to this receptor) [108,150]. Furthermore, these two enzymes play an important role during pregnancy in the regulation of active glucocorticoid concentrations [108]. The expression of these two enzymes, or the regulation of their activity, may be different between the H- and L-lines and remains to be investigated (especially during pregnancy).

### 3.4. Mechanism of Action of Glucocorticoids

The effectiveness of the HPAA to counter stress will influence energy metabolism and food intake, immune responses, behaviour, fertility and sexual libido, as well as the ability for learning in complex ways [84]. The mechanism of action and the main effects of glucocorticoids, the final product of HPAA, during stress will be discussed in the following paragraphs.

Glucocorticoids are lipophilic and can cross the cell membrane. However, 90% of glucocorticoids are transported in the blood, where they are bound to corticosteroid binding globulin (CBG) [151,152]. The remaining 10% of glucocorticoids are either free or bound to albumin. Free glucocorticoids readily diffuse across the cell membrane and exert their effects via intracellular receptors namely the glucocorticoid receptor (GR) [153]. GR is a cytosolic protein that is expressed in almost all tissue types [154,155]. The GR is maintained in the cytoplasm as an inactive multi-protein complex, where it is bound to heat shock protein 90. Binding of the ligand to GR induces a conformational change that results in the dissociation of the multi-protein complex, followed by the translocation of GR into the nucleus. The GR is able to bind to DNA sequences, known as glucocorticoid response elements (GREs), where it can either transactivate or transrepress the transcription of responsive genes [156,157]. There are different models for the molecular mechanisms by which the GR, as homodimers or GR monomer via protein-protein interaction with other transcription factors, interact with different types of GREs [158], but these models are beyond the scope of this discussion. 

This mechanism of action of the glucocorticoids allows for the regulation of the catabolic responses to stress, as well as non-stress related modulation of carbohydrate, protein and lipid metabolism. The effects of glucocorticoids on carbohydrate metabolism mostly involve the stimulation of gluconeogenesis and glycogen synthesis in the liver, while simultaneously increasing the substrate availability to these pathways by stimulating lipolysis and the release of glycogenic amino acids from peripheral tissues. Glucocorticoids stimulate gluconeogenesis in the liver by activating key enzymes, such as glucose-6-phosphatase, phosphoenolpyruvate, tyrosine aminotransferase and gamma-glutamyltransferase [159,160,161,162]. The availability of substrates for gluconeogenesis is increased by various mechanisms after exposure to increased levels of glucocorticoids. Glucose uptake and utilization by peripheral tissues is limited by the action of glucocorticoids on glucose transport into the cells [161]. The release of glycogenic amino acids from peripheral tissues is stimulated by glucocorticoids [158]. The sensitivity of tissues to glucagons is increased by the permissive effect of glucocorticoids. The sensitivity to catecholamines in lipolysis (adipose tissue) and lactate production (muscle) is also enhanced by glucocorticoids. Lipolysis is therefore acutely activated in adipose tissue by glucocorticoids. The free fatty acids from the triacylglycerols provide the energy for the production of glucose from glycerol [95,158]. Furthermore, glycogen synthesis in the liver is stimulated by the activation of glycogen synthase and the inactivation of glycogen phosphorylase by the action of glucocorticoids [163]. 

The autonomic nervous system and the HPAA interact in a complex manner that affects meat quality. The catecholamines typically alter energy metabolism by increasing lipolysis, glycogenolysis in the muscle and gluconeogenesis [164,165]. These energy-mobilizing effects of the catecholamines are amplified by glucocorticoids, as described in the previous paragraph. The relative effects of glucocorticoids on muscle protein metabolism are less clear than for catecholamines [166], and it is difficult to discriminate between their effects on meat quality during stressful events [165]. It is well known that chronic stress affects muscle glycogen depletion and the dark cutting condition [165]. The study by Jacob *et al*. [167] measured the average change in muscle glycogen in the *semitendinosus* and *semimembranosus* muscles of sheep between farm and slaughter at the abattoir. They demonstrated that the muscle glycogen, varied from negative to positive between consignments in relation to differences in their stress responsiveness [167]. Similarly, Cloete *et al*. [38] demonstrated that meat from “calmer” H-line sheep had a more vivid red colour and lower initial pH values (along with other characteristics) in comparison to L-line sheep.

Glucocorticoids also have a suppressive impact on immune function. As previously mentioned, glucocorticoids are transported in the blood bound to CBG, which is a member of the serine protease inhibitor super family. CBG is cleaved by serine protease elastase, which accumulates at sites of inflammation, and thereby promotes the release of glucocorticoids [168,169]. Glucocorticoids are thus released at such sites of inflammation where they can exert anti-inflammatory effects to minimize potential tissue damage. The innate immune response is altered by the action of glucocorticoids when it prevents the migration of leukocytes from blood circulation into extravascular fluids, decrease the number of circulating eosinophils and basophils, while increasing the blood counts of neutrophils, red blood cells and platelets [158,170,171,172]. Glucocorticoids down-regulate the synthesis and secretion of pro-inflammatory cytokines, such as interleukin-6 and interleukin-1β [173]. The cytokine-driven upregulation of some acute phase proteins are also enhanced by the action of glucocorticoids [174]. The acquired immunity response is suppressed by the action of glucocorticoids where the number of circulating lymphocytes is decreased. Glucocorticoids also inhibit the production of antibodies and the activity of helper T-cells and cytotoxic T-cells.

A selection programme where sheep were selected either for high (HCR) or low (LCR) cortisol responses to *Escherichia coli* endotoxin (endotoxemia) was undertaken to investigate which genetic factors within the HPAA and immune system contribute to variability within the cortisol response to endotoxemia [52]. It was concluded that the variability of cortisol responses to endotoxemia results from the variability within the HPAA function, and not the immune-based signalling events that lead to HPAA stimulation. Furthermore, substantial evidence has been provided that the levels of CBG may be an important genetic factor involved in the resistance to endotoxemia [175]. It is important to note that the anti-inflammatory action of glucocorticoids is beneficial on a short-term basis to fight infection and tissue damage, but elevated cortisol levels over an extended period of time will inhibit inflammatory processes to the extent that will eventually increase the animal’s susceptibility to pathogens [15,70].

Glucocorticoids have additional effects apart from energy metabolism and immunity. These include an increase in alertness and cognition, alteration in cardiovascular tone, an increase in blood pressure, increase in respiratory rate and an increase in bone resorption [95,103]. Glucocorticoids also inhibit the production and secretion of growth hormone and gonadotropin [84,147], which subsequently inhibits growth and reproduction. Murphy *et al*. [77], for example, reported that animals with a more quiet temperament grew faster and were better producers compared to nervous, restless and aggressive animals. This characteristic was observed for the “calmer” H-line sheep that had higher weaning weights [28] and live weights [30] than the more “nervous” L-line sheep. Another example is the disruption of preovulatory events by stress-like concentrations of cortisol that results in the impairment of follicular development and subsequent lower reproduction rate [176,177]. A similar mechanism may contribute to the lower reproductive rate observed for L-line sheep [21], compared to the H-line sheep, since the HPAA of L-line sheep is less responsive than the H-line [40,41,42] and subsequently the L-line animals would take longer to adapt to stressful situations. 

Furthermore, the deposition of glycogen stores in the foetus closer to term is essential for neonatal survival [178]. The glycogen stores serve as energy source to sustain metabolism until the establishment of a suckling. The increase in cortisol 10–15 days prior to parturition is crucial in preparing the foetus for extrauterine life. In addition, glucocorticoids are critical for the onset of parturition (via a glucocorticoid-prostaglandin feed-forward loop), where increased glucocorticoids primarily originate from the maturing foetal adrenal gland, rather than from maternal origin [107,178]. Preliminary results of H- and L-line embryos (unpublished data from 2009–2011 progeny [179], which were randomly implanted in recipient ewes in a multiple ovulation and embryo transfer (MOET) programme, indicated that H-line lambs are born approximately 1 day earlier than L-line lambs. Gestation lengths could be estimated within 4 hours of accuracy, due to control exerted through intra-uterine artificial insemination for MOET. This indicates that the higher HPAA responsiveness in adult H-line sheep [56] may translate to higher continual activation of the foetal HPAA in the H-line, which results in the earlier foetal maturation and onset of parturition than the L-line. As mentioned previously, H-line ewes gave birth 2.6 days earlier than L-line ewes under natural reproductive conditions [21], whereas H-line ewes also experienced shorter births [22]. These collective results suggest that both maternal and neonatal HPAA function may influence the gestation length and duration of parturition. 

Finally, glucocorticoids exert a negative feedback on the HPAA by acting on the pituitary, hypothalamus and higher levels in the central nervous system. This feedback action of glucocorticoids ensures the return of the HPAA activity to basal levels after stimulation. The hippocampus (part of limbic system) and PVN are the two brain centres with the highest density of glucocorticoid and mineralocorticoid receptors and are considered to be the main regulators of glucocorticoid feedback in the brain [180,181]. Only free corticosteroids are able to cross the blood-brain barrier and their concentrations thus determine the strength of the feedback, which ultimately inhibits CRH production and release [84]. Glucocorticoids also inhibit the production and release of ACTH from corticotrophs in the anterior pituitary. In addition, chronically elevated glucocorticoid levels can down-regulate the intracellular concentrations of their receptors. The negative-feedback of glucocorticoids to the HPAA is critical in preventing the detrimental effects of chronically elevated glucocorticoid concentrations. The expression of GR and mineralocorticoid receptor under different conditions remains to be investigated in the various tissues types of H- and L-line sheep. 

## 4. Conclusions

From this discussion it is clear that a whole array of factors contribute to variability of HPAA function. The identification of these factors holds great potential for MAS, since the HPA function affects animal health, welfare, production and reproduction in a complex manner. Potential genetic markers of HPAA function should, however, be assessed with great caution, with specific attention to whether acute or chronic HPAA function is being assessed. This may also involve careful consideration of the type of biological sampling. Generally, a chronic exposure to elevated cortisol concentrations is likely to have negative effects on animal production and reproduction. However, a rapid cortisol response on stimulation of the HPAA is beneficial to adapt to stressful situations and therefore has positive effects in relation to robustness-related traits. 

The example of the H- and L-lines that were selected for maternal multiple rearing ability has provided evidence that it is possible to make genetic progress in traits related to both animal welfare and production. It was demonstrated that some of the differences observed between the H- and L-lines may result from a difference in the HPAA function. It was possible to identify the potential mechanisms within the HPAA that may be involved in causing these differences. These mechanisms may then be used to speed up the process of identifying genetic factors with potential use in MAS. One such example was the identification of the two SNPs within the CYP17 genotype that contributes to HPAA responsiveness and behavioural responses to stress.

We conclude that the H- and L-line resource flock may be used as a model population for further studies on the HPAA function. Further studies may potentially lead to the formulation of guidelines for utilizing HPAA function to improve animal welfare and production simultaneously. We suggest that selection for factors that increase acute HPAA responsiveness, and selection against factors that contribute to chronically elevated cortisol levels, may ensure that high levels of animal welfare are maintained with a high level of productivity. 
